# Northampton General Hospital

**Published:** 1908-01-25

**Authors:** 


### Resident Medical Officers' Department.

### NORTHAMPTON GENERAL HOSPITAL.

### THE CORONER'S JURY AND THE MEDICAL OFFICERS.

On December 21, 1907, a man was brought to the Northampton General Hospital apparently suffering from the obscure results of an accident of which no clear account could be obtained. A very careful examination of the patient was made by two of the resident medical officers, but beyond the symptoms of shock no evidence of injury could be found. He was, however, detained under observation for an hour and a half, and at the end of that time, since nothing further transpired, he was sent home in a cab. But, as was found later, there were serious internal injuries, and the man died from them soon after reaching home. Such mistakes in the diagnosis of obscure conditions are bound to occur from time to time, even when the greatest skill and care are exercised. They are regrettable, but they cannot always be avoided. In the present instance it is clear that considerable trouble was taken by the medical officers, and that they formed their opinion only after a thorough and deliberate examination.

In view of these facts the action of the coroner's jury in charging the medical staff with inhumanity is greatly to be deplored. The Hospital Board immediately made the fullest inquiries into all the circumstances of the case, and have published the result of their investigations. They state that everything that possibly could have been done was done, and that the medical officers showed the greatest skill and kindness. They are, therefore, of the opinion that the charge of inhumanity is without foundation, and this belief is fully borne out by the result of the post-mortem examination.

It is pleasant to find that these unfounded accusations on the part of the coroner's jury are not endorsed by the leading local newspapers. The *Northampton Mercury* devotes an able leader to the vindication of the resident medical officers, and incidentally to an appreciation of the medical profession. This article is admirable in style, and just and temperate in tone. We hope that the plain statement of the Hospital Board and the comments of the influential local press will atone in some degree to the medical officers for the public indignity to which they have been subjected.

